# Hepatitis B Virus Reactivation in Kidney Transplant Recipients Treated With Belatacept

**DOI:** 10.1016/j.ekir.2023.05.005

**Published:** 2023-05-16

**Authors:** Chloë Schwarz, Antoine Morel, Marie Matignon, Philippe Grimbert, Eric Rondeau, Nacera Ouali, Hélène François, Laurent Mesnard, Camille Petit-Hoang, Cédric Rafat, Karine Dahan, Yosu Luque

**Affiliations:** 1 Soins Intensifs Néphrologiques et Rein Aigu, Département de Néphrologie, Hôpital Tenon, Assistance Publique–Hôpitaux de Paris, Paris, France; 2 Service de Néphrologie, Hôpital Henri Mondor, Assistance Publique–Hôpitaux de Paris, Créteil, France; 3 Sorbonne Université, Unité CoRaKid, Inserm, UMR_S1155, Paris, France; 4 Néphrologie et Dialyses, Département de Néphrologie, Hôpital Tenon, Assistance Publique–Hôpitaux de Paris, Paris, France

**Keywords:** Belatacept, HBcAb-positive, HBV reactivation, Hepatitis B virus, kidney transplantation

## Abstract

**Introduction:**

Hepatitis B virus (HBV) reactivation in kidney transplant recipients has been reported in 3% to 9% of anti-HBc antibody (HBcAb)-positive HBs antigen (HBsAg)-negative patients. It has not been studied in patients receiving belatacept, a selective costimulation blocker.

**Methods:**

We performed a retrospective study of all transplant recipients receiving belatacept in 2 kidney transplantation centers in France. Among HBcAb-positive patients, we analyzed HBV reactivation rate, outcomes, and risk factors.

**Results:**

A total of 135 patients treated with belatacept were included: 32 were HBcAb-positive and 2 were HBsAg-positive. Seven patients reactivated HBV (21.9% of HBcAb-positive patients), including 5 HBsAg-negative patients (16.7% of HBcAb-positive HBsAg-negative patients). Reactivation occurred 54.8 (± 70.9) months after transplantation. One patient presented with severe hepatitis and 1 patient developed cirrhosis. There was no significant difference in survival between patients that reactivated HBV and patients that did not: 5-year patient survival of 100% (28.6; 100) and 83.4% (67.6; 100), respectively (*P* = 0.363); and 5-year graft survival of 100% (28.6; 100) and 79.8% (61.7; 100), respectively (*P* = 0.335). No factor, including HBsAb positivity and antiviral prophylaxis, was statistically associated with the risk of HBV reactivation.

**Conclusion:**

HBV reactivation rate was high in patients treated with belatacept when compared with previous transplantation studies. HBV reactivation did not impact survival. Further studies are needed to confirm these results. A systematic antiviral prophylaxis for these patients should be considered and evaluated.

## Graphical abstract

**Figure:**
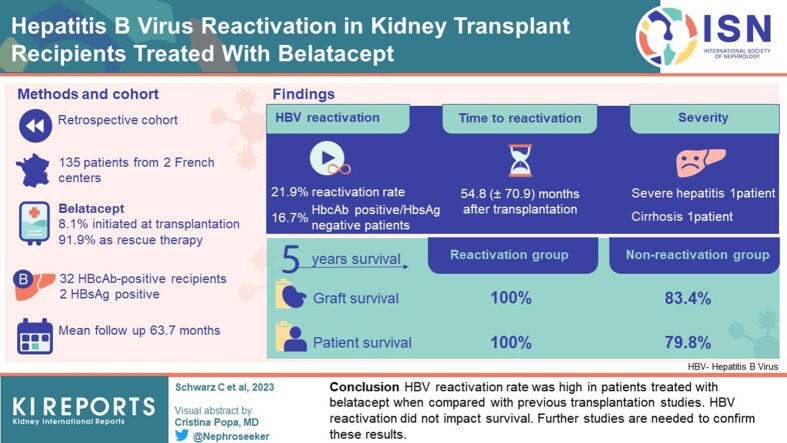

Infection with HBV is a global public health burden. Its prevalence is high; approximatively 250 million people are HBV surface antigen (HBsAg) carriers worldwide, with significant variation across countries.^1^ Its severity is due to the development of cirrhosis and hepatocellular carcinoma. After contact with the virus, patients develop anti-hepatitis B core antigen antibodies (HBcAb), indicating exposure to HBV. Infection can be resolved if the host’s immunologic response is rapid and efficient, in which case anti-HBs antibodies (HBsAb) develop. On the contrary, chronic HBV infection is defined by the presence of HBsAg for at least 6 months.^2^

Patients with end-stage kidney disease are at higher risk of HBV infection than the general population. Prevalence of HBV infection in kidney transplant recipients is therefore high: positivity of HBsAg is estimated at 1.4% in this population in France,^3^ and positivity of HBcAb ranges from 7.9% in the USA^4^ to 16.3% in Germany,^5^ and 20% in Japan.^6^

Historically, HBsAg-positive patients displayed worse outcomes following kidney transplantation.^7^, ^8^, ^9^ For instance, in a meta-analysis of 6050 patients by Fabrizi *et al.*,^8^ HBsAg positivity at time of transplantation was associated with a relative risk of death of 2.49 compared to HBsAg-negative patients. However, more recent studies do not seem to concur with these results,^3^ suggesting that prognosis improved with more generalized use of antiviral therapy.

HBV reactivation in immunocompromised patients has been described in various clinical settings. It is especially well-documented for patients treated with B-cell depleting agents such as rituximab, for which the risk is considered the highest (16.9% in a pooled analysis of 325 patients).^10^^,^^11^ Risk of HBV reactivation concerns HBsAg-positive patients as well as HBsAg-negative patients who are HBcAb-positive, and can be asymptomatic or take the form of fulminant hepatitis; in the long-term it is associated with the development of cirrhosis. In the kidney transplant recipient population, risk of HBV reactivation has not been extensively studied; it ranges from 0% to 9.6% of HBsAg-negative patients who are HBcAb-positive according to different retrospective cohort studies.^12^, ^13^, ^14^, ^15^, ^16^, ^17^, ^18^, ^19^ A few cases of severe hepatitis were reported^13^, ^14^, ^15^ and the results are conflicting concerning the impact of HBV reactivation on patient and graft survival.^13^^,^^14^ Recommendations concerning prophylaxis and treatment of HBV reactivation in this population are scarce and only relate to HBsAg-positive patients or patients receiving rituximab.^20^

Belatacept is a selective costimulation blocker used in kidney transplantation. It binds to the surface ligands CD80 and CD86 of antigen-presenting cells and prevents their interaction with the surface costimulatory receptor CD28 of T cells, thus inhibiting their full activation.^21^ Generally used in association with corticosteroids and mycophenolate mofetil, its main interest is to replace calcineurin inhibitors, therefore avoiding their toxicity. Its effectiveness in preventing rejection has been demonstrated.^22^^,^^23^ Safety was also established, even though concerns were raised regarding viral infections. Indeed, a higher occurrence of Epstein-Barr virus (EBV) related posttransplant lymphoproliferative disorder was noticed.^24^^,^^25^ In addition, cases of progressive multifocal leukoencephalopathy associated with JC virus were reported.^24^^,^^26^ Concerning cytomegalovirus (CMV) infections, 4 studies showed high rates of CMV replication^27^^,^^28^ and CMV symptomatic infections.^26^^,^^29^ Finally, frequent BK viremia and BK virus nephropathy have been reported by several studies.^27^^,^^30^

No data exist pertaining to the risk of HBV reactivation when undergoing treatment with belatacept in the kidney transplantation setting, except for 1 case report.^31^ Here, we conducted a retrospective cohort study of all kidney transplant recipients receiving belatacept in 2 transplantation centers in France. The objective was to quantify the risk of HBV reactivation in HBcAb-positive patients, to explore whether patients reactivating HBV had poorer outcomes, and to identify risk factors for HBV reactivation.

## Methods

This retrospective cohort study was conducted in 2 transplantation centers in France: Tenon hospital, Paris and Mondor hospital, Créteil. The patients underwent kidney transplantation between July 1993 and July 2019.

### Patients

All patients that underwent kidney transplantation and received belatacept were included, whether belatacept was prescribed at transplantation or after calcineurin inhibitors were stopped. Patients were transplanted from deceased or living donors and could receive combined transplants. If a patient had received more than 1 transplant, these were included as individual episodes.

When started at transplantation, belatacept was administered intravenously at a dose of 10 mg/kg on days 1, 5, 14, 28, 56, and 84; and then at 5 mg/kg every 4 weeks. When started later, it was administered at a dose of 5 mg/kg every 2 weeks for the first 5 injections and then every 4 weeks. There was no minimal duration of treatment.

### Data Collection

All patients’ medical files were retrospectively examined. All data were recorded anonymously. Data at baseline were collected regarding the following:

• Initial characteristics include age, sex, geographic origin, etiology of renal disease, prior kidney transplantation, prior immunosuppressive treatment, duration of dialysis, HBV serologic status (HBsAg, HBcAb, and HBsAb), as well as HIV, hepatitis C virus, EBV and CMV serologic statuses.
• Characteristics of the transplant include date of transplantation, type and age of donor, assessment of immunologic risk (hyperimmunization defined as panel-reactive antibodies >85%, presence of donor-specific antibodies at time of transplantation and number of human leukocyte antigen A/B/DR/DQ mismatches), as well as induction and maintenance immunosuppressive regimen.

The following data on the outcome of transplantation were collected during follow-up: occurrence of a biopsy-proven acute rejection episode, death and graft loss as well as estimated glomerular filtration rates (eGFR) at 3 months, 1 year, and 5 years posttransplantation. eGFR was analyzed for patients with functioning grafts only and the Modification of Diet in Renal Disease formula was used. Graft loss was defined as return to dialysis or retransplantation, and was censored for death. Data concerning replication of CMV, EBV, and BK virus were also registered, viral replication being defined as a positive polymerase chain reaction (PCR).

Data concerning HBV included the number of serologic tests and HBV PCRs performed during follow-up, occurrence of HBV reactivation (defined as *de novo* HBV PCR >1 log), positivity of HBsAg and occurrence of acute hepatitis (defined by alanine aminotransferase levels >3 times the high rank of the normal level). Data on HBV antiviral therapy were also recorded. Finally, loss of HBsAb, defined as a titer of HBsAb <10 IU/l observed in a previously HBsAb-positive patient and without further repositivation, was registered.

Length of follow-up was recorded. Patients were followed until death or repeat transplantation. Follow-up was ended in August 2019.

### Study End Points

The primary objective of the study was to describe HBV reactivation and report HBV reactivation rate among belatacept-treated kidney transplant recipients with positive HBcAb. We also analyed the following:

• Patient and graft survival in the whole cohort and according to HBcAb status.
• Impact of HBV reactivation on patient and graft survival among HBcAb-positive patients.
• Risk factors associated with HBV reactivation.
• Occurrence of other viral reactivations.

### Ethics Statement

The study was conducted in accordance with the ethical guidelines of the Assistance Publique–Hôpitaux de Paris. No institutional review board approval was necessary at the time of the study because it was a retrospective study involving no intervention. The study was conducted according to the ethical standards of the 2000 Declaration of Helsinki as well as the 2008 Declaration of Istanbul.

### Statistical Analysis

Quantitative data are presented as mean ± SD. Categorial data are presented as numbers and percentages. Survival data were analyzed with Kaplan-Meier estimator and survival between groups was compared using the log-rank test. When comparing quantitative data with normal distribution, Student’s t-test was used; if data did not follow a normal distribution, Wilcoxon-Mann-Whitney test was used. When comparing qualitative data (percentages), Pearson’s χ^2^ test was used. Among HBcAb-positive patients, factors associated with HBV reactivation were investigated using univariate logistical regression.

A *P-*value of <0.05 was considered statistically significant. All statistical tests were performed using the R software (version 2020, R Foundation for Statistical Computing, Vienna, Austria. https://www.R-project.org/).

## Results

### Cohort Characteristics

**Figure 1:**
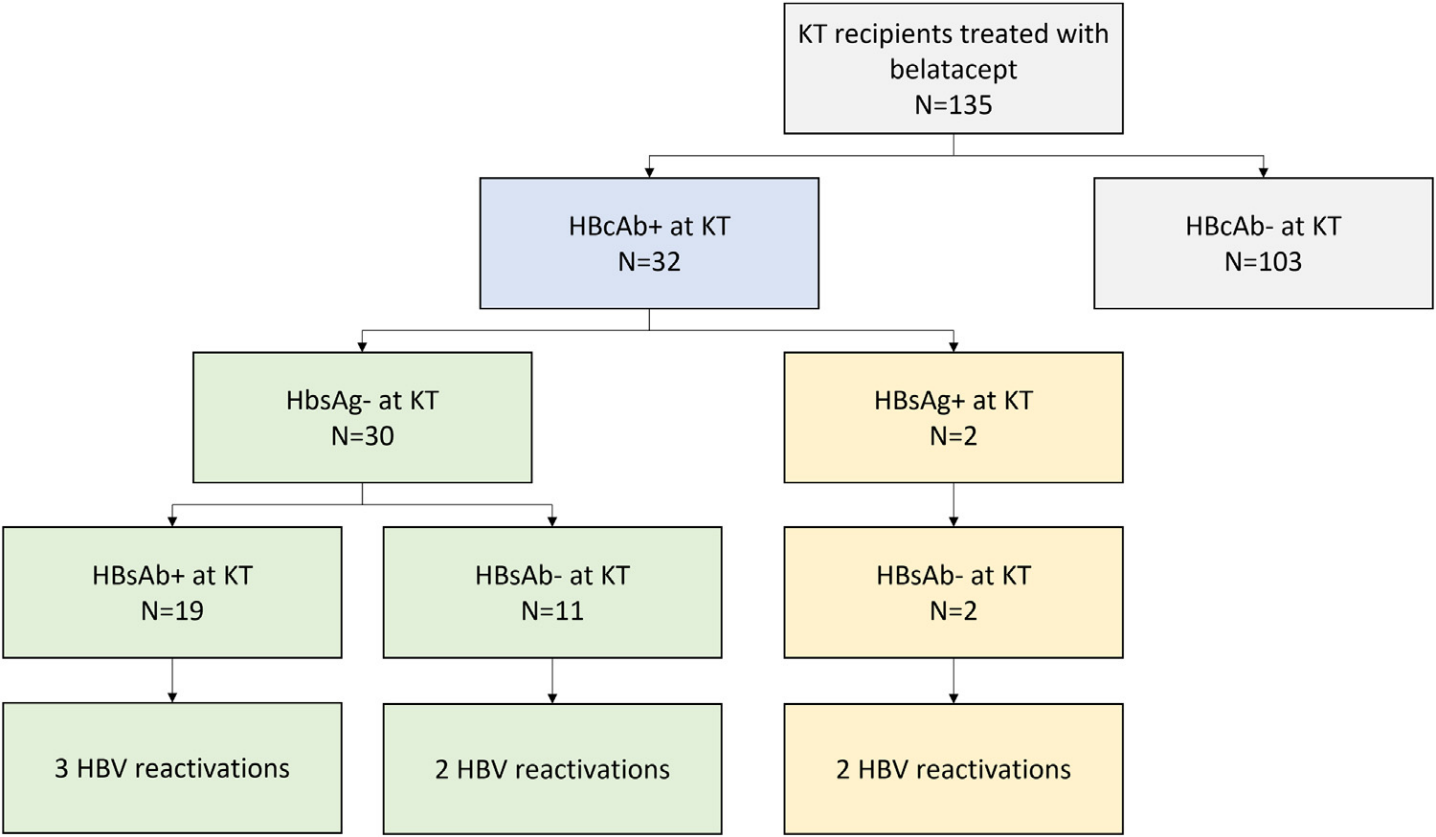
Patient distribution and HBV reactivation according to HBcAb, HBsAg, and HBsAb status. KT, kidney transplantation; HBcAb−, negative for anti-HBc antibodies; HBcAb+, positive for anti-HBc antibodies; HBsAg−, negative for HBs antigen; HBsAg+, positive for HBsAg antigen; HBsAb−, negative for HBs antibodies; HBsAb+, positive for HBs antibodies.

A total of 135 patients were included in the study. The study’s flowchart is presented in Figure 1. For 11 patients (8.1%), belatacept was initiated at transplantation. For the other 124 patients (91.9%), belatacept was started as a rescue strategy because of calcineurin inhibitors toxicity and/or low graft function in 97 cases, because of thrombotic microangiopathy evidence in 19 cases, and for another or unspecified cause in 8 cases. Mean time from transplantation to belatacept initiation was 31.9 (±51.6) months.

Thirty-two patients were HBcAb-positive, which represented 23.7% of the total cohort. Of these 32 patients, 30 were HBsAg-negative at the time of transplantation and 2 were HBsAg-positive, suggesting chronic HBV infection. All patients had negative HBV PCR at the time of transplantation.

The demographic and clinical characteristics of all patients, HBcAb-positive patients and HBcAb-negative patients, are summarized in Table 1. Eight patients (5.9%) were HIV-positive and 4 patients (3.0%) were hepatitis C virus-positive. Twenty-eight patients (20.7%) received immunosuppressive therapy before transplantation. Induction therapy was thymoglobulin in 52.6 % of cases and basiliximab in 47.4% of cases. Of the patients, 130 (96.3%) received mycophenolate mofetil, 125 (92.6%) received calcineurin inhibitors, 35 (25.9%) received mammalian target of rapamycin inhibitors. All patients received corticosteroids. At time of transplantation or during follow-up, 13 patients (9.7%) were administered rituximab. Of the patients, 35 (25.9%) were treated for a biopsy-proven acute rejection episode. Mean length of follow-up was 63.7 (±54.0) months.

**Table 1:** Demographic and clinical characteristics of patients

Characteristics	All patients (*n* = 135)	HBcAb+ patients (*n* = 32)	HBcAb-patients (*n* = 103)	*P*-value
Age at KT (yr)	53.8 (± 15.9)	55.2 (± 15.6)	53.4 (± 16.0)	0.566
Female	49 (36.3 %)	9 (28.1 %)	40 (38.8 %)	0.271
Geographic origin				
Sub-Saharan Africa/Caribbean	34 (25.2 %)	18 (56.2 %)	16 (15.5 %)	
North Africa	18 (13.3 %)	2 (6.2 %)	16 (15.5 %)	
Europe	45 (33.3 %)	5 (15.6 %)	40 (38.8 %)	
Asia	7 (5.2 %)	4 (12.5 %)	3 (2.9 %)	
America	2 (1.5 %)	0 (0 %)	2 (1.9 %)	
Initial nephropathy				
Diabetes	17 (12.6 %)	2 (6.3 %)	15 (14.6 %)	
Nephroangiosclerosis	10 (7.4 %)	4 (12.5%)	6 (5.8 %)	
Chronic glomerulonephritis	26 (19.3 %)	8 (25.0 %)	18 (17.5 %)	
Systemic diseases	11 (8.1 %)	0 (0 %)	11 (10.7 %)	
ADPKD	10 (7.4 %)	1 (3.1 %)	9 (8.7 %)	
Other etiologies	29 (21.5 %)	5 (15.6 %)	24 (23.3 %)	
Unknown	32 (23.7 %)	12 (37.5 %)	20 (19.4 %)	
Length of dialysis (mo)	38.5 (± 32.9)	44.4 (± 25.6)	36.65 (± 34.8)	0.614
HBsAb+	98 (72.6 %)	19 (59.4 %)	78 (76.5 %)	0.055
HCV+	4 (3.0 %)	2 (6.3 %)	2 (1.9 %)	0.209
HIV+	8 (5.9 %)	6 (18.8 %)	2 (1.9 %)	0.0004
2nd or 3rd KT	18 (13.3%)	2 (6.3 %)	16 (15.5 %)	0.177
Living donor	11 (8.1 %)	1 (3.1 %)	10 (9.7 %)	0.234
Age of donor	59.9 (± 15.1)	60.8 (± 15.3)	59.58 (± 15.07)	0.680
Immunosuppressive therapy before KT	28 (20.7%)	5 (15.6%)	23 (22.3%)	0.374
Hyperimmunised status (PRA > 85%)	16 (11.8 %)	2 (6.3 %)	14 (13.6 %)	0.249
HLA A/B/DR/DQ mismatches	4.5 (± 1.7)	5.1 (± 1.4)	4.31 (± 1.74)	0.017
DSA at KT	37 (27.4 %)	14 (43.8 %)	23 (22.3 %)	0.018
Thymoglobulin as induction therapy	71 (52.6 %)	19 (59.4 %)	52 (50.5 %)	0.407
Rituximab treatment (any time)	13 (9.6 %)	3 (9.4 %)	10 (9.7 %)	0.943
Acute rejection episode	35 (25.9 %)	6 (18.8 %)	29 (28.2 %)	0.277
Time from KT to belatacept (mo)	29.3 (± 50.2)	24.1 (± 51.9)	30.9 (± 49.8)	0.140
Length of follow-up (mo)	63.7 (± 54.1)	59.8 (± 55.9)	64.9 (± 53.7)	0.439

ADPKD, autosomal dominant polycystic kidney disease; DSA, donor-specific antibodies; HBcAb+, positive anti-HBc antibodies; HBcAb−, negative anti-HBc antibodies; HBsAb+, positive anti-HBs antibodies; HCV+, positive HCV serology; HIV+, positive HIV serology; HLA, human leukocyte antigen; KT, kidney transplantation; PRA, panel-reactive antibodies.

Numeric continuous values are presented as mean (±SD) and categorial variables as n (percentage).

Demographic and initial clinical characteristics were generally comparable between HBcAb-positive and HBcAb-negative patients. Of note, HIV seropositivity was more frequent in HBcAb-positive patients (18.8% vs. 1.9%, *P* = 0.0004). HBcAb-positive patients also presented more donor-specific antibodies at transplantation (43.8% vs. 22.3%, *P* = 0.018).

### Patient and Graft Survival According to HBcAb Status

**Figure 2:**
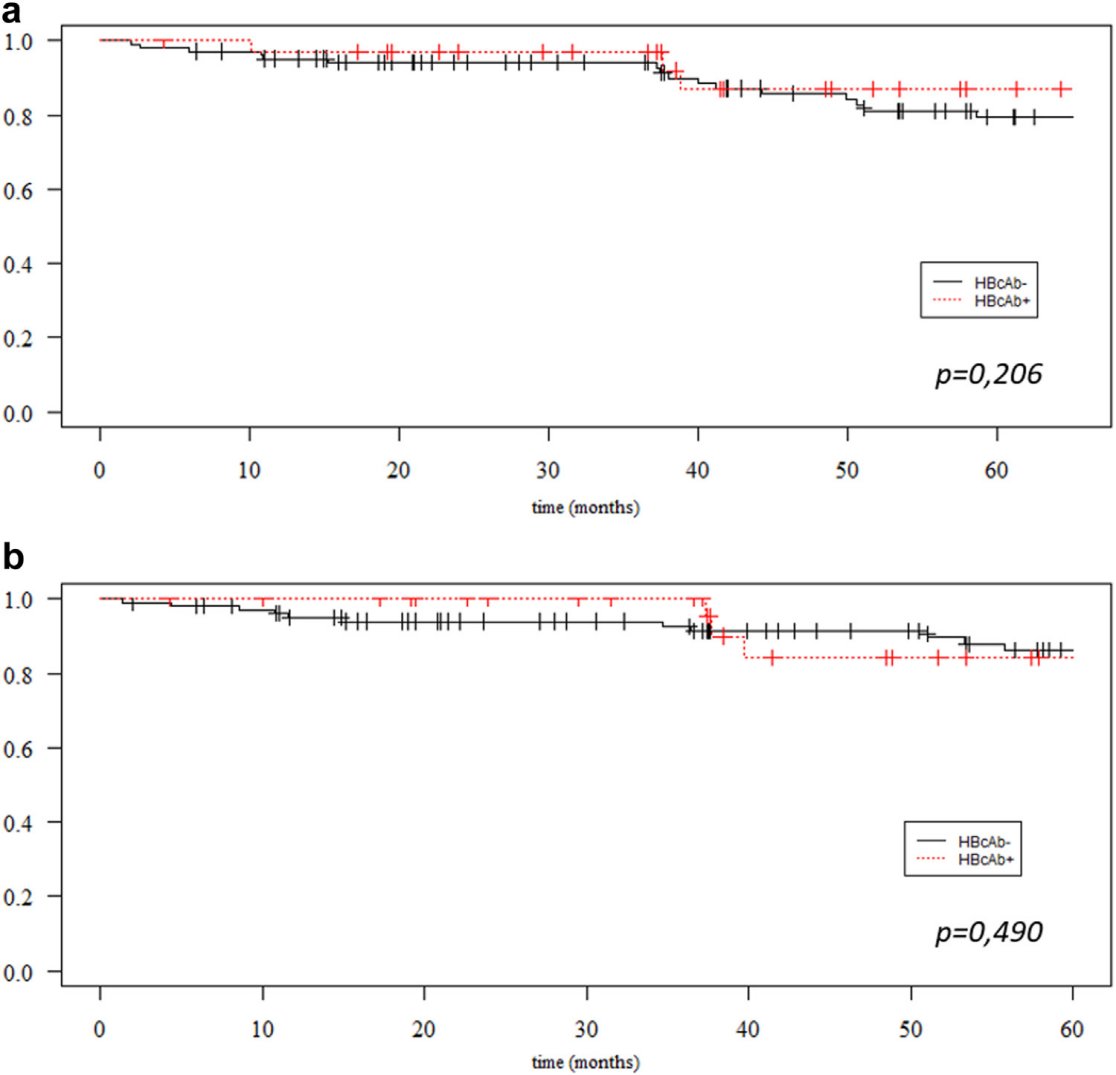
Patient and graft survival according to HBcAb status. Kaplan-Meier survival curves for patients (a) and graft (b) survival according to HBcAb status at time of transplantation. HBcAb−, negative for anti-HBc antibodies; HBcAb+, positive for anti-HBc antibodies. Statistical difference was assessed with the log-rank test.

Among all patients treated with belatacept, patient survival rates were 95.5% (95% confidence interval [CI]: 92.0; 99.1) at 1 year, and 80.7% (95% CI: 73.0; 89.2) at 5 years. During the follow-up period, a total of 26 patients died; 11 deaths were related to infection, 4 to cancer, 3 to cardiovascular events, and in 8 cases the cause was unknown. There was no significant difference in patient survival between HBcAb-positive (*n* = 32) and HBcAb-negative patients (*n* = 103), (*P* = 0.206). Indeed, at 5 years, patient survival in HBcAb-positive and HBcAb-negative groups was 86.8% (95% CI 73.7; 100) and 79.3 % (95% CI 70.4; 89.2), respectively (Figure 2a).

Among all patients, death-censored graft survival rates were 96.2% (95% CI: 92.9; 99.5) at 1 year, and 85.7% (95% CI: 80.7; 94.2) at 5 years. There was no significant difference between the 2 groups (*P* = 0.490) with a 5-year graft survival of 84.3% (95% CI: 69.4; 100) for HBcAb-positive patients and of 86.1% (95% CI: 78.5; 94.4) for HBcAb-negative patients (Figure 2b). Likewise, 1-year eGFR was similar in HBcAb-positive and HBcAb-negative groups with a mean of 44.2 (±16.5) and 42.0 (±16.9) ml/min per 1.73 m^2^ respectively, *P* = 0.552. In contrast, 5-year eGFR appeared significantly different between the 2 groups, with a mean of 54.2 (±18.4) for HBcAb-positive patients and of 40.1 (±14.9) ml/min per 1.73 m^2^ for HBcAb-negative patients, (*P* = 0.0213).

### Characteristics and Management of HBcAb-positive Patients

Two of the 32 HBcAb-positive patients had positive HBsAg at the time of transplantation. Nineteen (59.4%) had positive HBsAb at the time of transplantation; the mean HBsAb titer for these patients was 275 (± 307) UI/l. Ten patients (31.3%) received HBV antiviral prophylaxis after kidney transplantation; 7 received entecavir, 2 received lamivudine and 1 received tenofovir (the latter was part of the patient’s HIV antiviral therapy). Among these 10 patients were the 2 HBsAg-positive patients. During follow-up, a mean of 1.3 (±1.5) PCR per patient and per year of follow-up was performed. Similarly, a mean of 5.3 (±4.7) HBV serologic tests were performed per patient, or 1.2 (±1.1) per patient and per year of follow-up. Of note, no HBV PCR at any time during follow-up was performed for a total of 6 HBcAb-positive patients (18.8%).

### Description of HBV Reactivations

Seven patients experienced HBV reactivation (21.9% of HBcAb-positive patients). When focusing on the 30 HBsAg-negative HBcAb-positive patients, 5 experienced HBV reactivation (16.7%) (Figure 1).

The data regarding the 7 HBV reactivations are summarized in Table 2. None of the 7 patients that reactivated HBV had received a previous kidney transplant. Two were coinfected with HIV. Six received thymoglobulin and 1 received basiliximab as induction therapy. None received rituximab and none experienced an acute rejection episode. Three patients received HBV antiviral prophylaxis before reactivation; entecavir (*n* = 2) and tenofovir as part of HIV therapy (*n* = 1); among these were the 2 HBsAg-positive patients. At the time of transplantation, 3 patients had positive HBsAb at protective titers (>10 IU/l) and only in 1 case did HBsAb become negative before reactivation.

**Table 2:** Description of the 7 HBV reactivation cases

Date of KT	Age	Induction therapy	HIV status	HBsAg at KT	HBsAb at KT	Loss of HBsAb	HBV antiviral prophylaxis	Time to reactivation (months)	Peak HBV PCR	Hepatitis (peak ALT)	Antiviral therapy after reactivation	HBV DNA clearance	Outcome (length of follow-up)
2016	59	ATG	Negative	Negative	0		None	20.4	5.22	No	Entecavir	Yes	Alive with functioning graft (37 mo)
2016	25	ATG	Negative	Positive	0		Entecavir	12.8	2.25	No	Entecavir	Yes	Alive with functioning graft (36 mo)
2015	45	ATG	Positive	Negative	312	Yes	None	25.5	7.0	Yes ALT 1857	Entecavir	Yes	Alive with functioning graft (51 mo)
2016	51	ATG	Negative	Negative	374	No (min 175)	None	32.0	1.0	No	None	Yes	Alive with functioning graft (37 mo)
2013	46	ATG	Positive	Positive	0		Tenofovir	56.5	8.26	No	Tenofovir	No	Cirrhosis Alive with functioning graft (72 mo)
2001	39	Anti-rIL2	Negative	Negative	0		None	212.6	7.43	No	Entecavir	Yes	Alive with functioning graft (221 mo)
2014	77	ATG	Negative	Negative	37	No (min 19)	Entecavir	23.9	2.68	No	Entecavir	Yes	Alive with functioning graft (57 mo)

ATG, thymoglobulin; Anti-rIL2, basiliximab; ALT, alanine aminotransferase; KT, kidney transplantation.

Age is expressed in years, HBsAb titers are expressed in IU/l, HBV PCR in log and ALT titers in IU/l (normal < 32 IU/l).

HBV reactivation occurred at a mean time of 54.8 (±70.9) months after transplantation and of 30.5 (±14.7) months after belatacept initiation. Acute hepatitis was observed in only 1 reactivation, with alanine aminotransferase titers increasing until 58 times over the normal range, without evidence of liver failure. Positivity of HBV PCR was accompanied by positivity of HBsAg in 5 out of the 7 cases. After reactivation, 6 patients received HBV antiviral therapy; 5 patients received entecavir (including the 2 patients who were already treated with the drug) and tenofovir was continued for the HIV-positive patient who had already received the drug. In the last case, HBV viral load spontaneously became negative on control 1 month later and no treatment was introduced. On HBV reactivation, mycophenolate mofetil was temporarily discontinued in 2 patients and then resumed when viral load diminished. During follow-up, HBV PCR became negative in 6 of the 7 patients. The last patient (who was treated with tenofovir and was HBsAg-positive at transplantation) did not clear HBV viral load; the virus was resistant to entecavir and lamivudine, and they eventually developed cirrhosis.

Among the 7 patients, 6 experienced CMV replication at one point during follow-up; one of them developed CMV disease concomitantly with HBV reactivation. Three patients presented BK virus replication in urinary samples; 1 patient had a biopsy-proven BK virus nephritis and it occurred at the same time as HBV replication. Six patients replicated EBV and 5 of them had a positive EBV viral load at the time of HBV reactivation.

After HBV reactivation, all patients were alive and with functioning grafts until the last follow-up; none experienced acute rejection.

### Factors Associated With HBV Reactivation

Among HBcAb-positive patients, the following risk factors for HBV reactivation were studied: age equal to or greater than 65 years at transplantation, male sex, HIV coinfection, prior immunosuppressive therapy, prior kidney transplantation, presence of donor-specific antibodies at transplantation, thymoglobulin as induction therapy, treatment with rituximab, occurrence of an acute rejection episode, HBsAb positivity at transplantation, and absence of HBV antiviral prophylaxis. After univariate logistic regression analysis, no factor showed significant association with HBV reactivation (Table 3).

**Table 3:** Univariate logistic regression analysis of factors associated with HBV reactivation

Variable	HBV reactivation	No HBV reactivation	Odds ratio	*P-*value
Age ≥65 yr at KT	1 (14.3%)	8 (32.0%)	0.35	0.362
Male sex	5 (71.4%)	18 (72.0%)	0.97	0.976
HIV coinfection	2 (28.6%)	4 (16.0%)	2.10	0.458
Prior immunosuppressive therapy	1 (14.3%)	4 (16.0%)	0.86	0.912
Prior KT	0 (0.0%)	2 (8.0%)	<0.01	0.995
Presence of DSA at KT	4 (57.1%)	10 (40.0%)	2.00	0.424
Induction with ATG	6 (85.7%)	13 (52.0%)	5.54	0.137
Treatment with rituximab	0 (0.0%)	3 (12.0%)	<0.01	0.994
Acute rejection episode	0 (0.0%)	6 (24.0%)	<0.01	0.995
HBsAb positivity at KT	3 (42.9%)	16 (64.0%)	0.42	0.321
No HBV antiviral prophylaxis	4 (57.1%)	18 (72.0%)	0.52	0.458

ATG, thymoglobulin; DSA, donor-specific antibodies; HBsAb, HBs antibodies; HBV, hepatitis B virus; KT, kidney transplantation.

Data are presented as mean (±SD) or n (percentage) in each group and as odds ratio with the correspondent *P*-value.

### Influence of HBV Reactivation on Patient and Graft Survival

**Figure 3:**
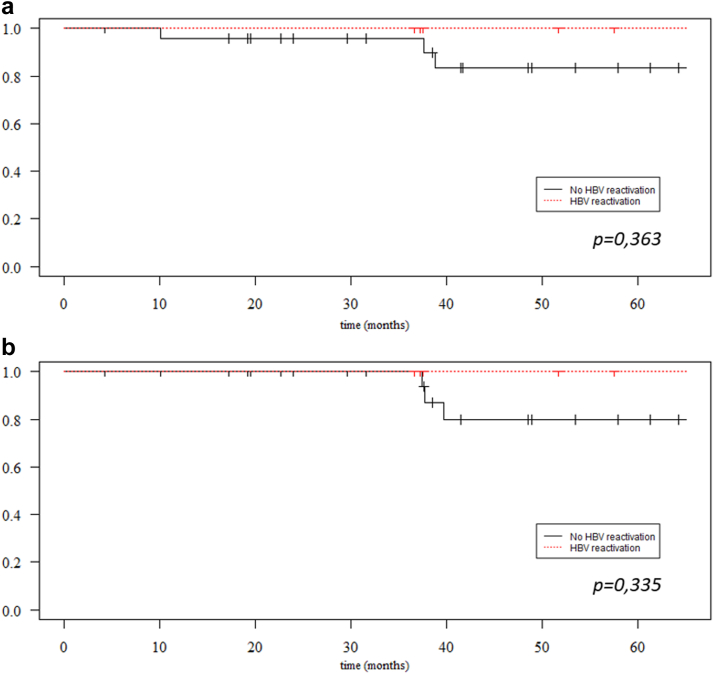
Patient and graft survival according to HBV reactivation. Kaplan-Meier survival curves for (a) patients and (b) graft survival according to HBV reactivation among HBcAb-positive patients. Statistical difference was assessed with the log-rank test.

Survival was compared between HBcAb-positive patients that reactivated HBV (*n* = 7) and HBcAb-positive patients that did not reactivate HBV (*n* = 25) during follow-up. Patient survival was not significantly different between the 2 groups of patients (*P* = 0.363). At 5 years, patient survival rates among patients with HBV reactivation and in patients free from HBV reactivation were 100% (95% CI: 28.6; 100) and 83.4% (95% CI: 67.6; 100), respectively (Figure 3a). Likewise, graft survival did not differ between the 2 groups of patients (*P* = 0.335), with a 5-year graft survival of 100% (95% CI: 28.6; 100) among patients with HBV reactivation and of 79.8% (95% CI: 61.7; 100) among patients free from HBV reactivation (Figure 3b).

### Loss of HBsAb in the Entire Cohort

Among the 135 patients that received belatacept, 98 (72.6%) were HBsAb-positive at the time of transplantation, with a mean HBsAb titer of 344 (±383) IU/l. At least 1 HBV serologic test was performed during follow-up for 86 (87.8%) of these HBsAb-positive patients and 18 became HBsAb-negative (20.9%). Loss of HBsAb occurred at a mean time of 29.3 (±34.0) months after transplantation.

Nineteen patients had a serologic profile of resolved HBV infection (HBcAb-positive and HBsAb-positive) accounting for 59.4% of HBcAb-positive patients. They had a mean HBsAb titer of 275 (±307) IU/l. Among them, 3 patients (15.8%) became HBsAb-negative during follow-up, which was associated with HBV reactivation in 1 patient; in that case, loss of HBsAb and HBV reactivation occurred concurrently. There was no significant association between loss of HBsAb and HBV reactivation (odds ratio: 3.50, *P* = 0.384 using univariate logistic regression analysis).

### Other Viral Replications

Among all patients, 105 (77.8%) were seropositive and 30 (22.2%) were seronegative for CMV at the time of transplantation. Among CMV-positive patients, 62 (59.0%) reactivated CMV at one point during follow-up, at a mean time of 6.1 (±6.1) months after transplantation. Among CMV-negative patients, 14 (46.7%) presented a primary infection at a mean time of 8.6 (± 5.7) months after transplantation. In total, 76 patients (56.3%) replicated CMV with a mean peak PCR of 3.78 (±1.14) log.

Among all patients, 130 (96.3%) were seropositive and 4 (3.0%) were seronegative for EBV at the time of transplantation. All the 4 EBV-negative patients replicated EBV at a mean time of 50.2 (±53.5) months after transplantation. One EBV-negative patient presented with EBV-related posttransplant lymphoproliferative disorder, which occurred 19 years after transplantation and 2 years after belatacept introduction; he was treated with 4 injections of rituximab and fully recovered. Among EBV-positive patients, 80 (62.0%) reactivated EBV at a mean time of 24.6 (±21.9) months after transplantation. One patient in this group also developed a posttransplant lymphoproliferative disorder 13 months after transplantation; belatacept was discontinued along with mycophenolate mofetil and he received rituximab and chemotherapy; renal graft was lost 2 months following diagnosis. In total, 85 patients (63.0%) replicated EBV with a mean peak PCR of 3.90 (±0.82) log and 2 posttransplant lymphoproliferative disorders occurred.

During follow-up, 46 patients (34.1%) presented with BK virus viruria at a mean time of 14.3 (±17.7) months after transplantation and 19 (14.1%) presented with BK virus viremia, at a mean time of 10.1 (±10.9) months after transplantation.

A comparison of CMV, EBV, and BK virus replication in all patients; in HBcAb-positive patients that did not reactivate HBV; and in HBcAb-positive patients that reactivated HBV is presented in Table 4.

**Table 4:** CMV, EBV, and BK virus replication in different groups of patients

Virus	Entire cohort *n* = 135	HBcAb+. no HBV reactivation *n* = 25	HBcAb+. HBV reactivation *n* = 7
CMV replication	76 (56.3%)	20 (80%)	6 (85.7%)
EBV replication	85 (63.4%)	16 (64%)	6 (85.7%)
BKv replication (urine)	46 (34.6%)	9 (36%)	3 (42.9%)
BKv replication (plasma)	19 (14.3%)	3 (12%)	1 (14.3%)

BKv, BK virus; CMV, cytomegalovirus; EBV, Epstein-Barr virus; HBcAb+, patients with anti-HBc antibodies.

Viral replication is defined by a positive viral load at any time during follow-up.

Data are expressed as n (percentage).

## Discussion

In this retrospective cohort including 32 HBcAb-positive kidney transplant recipients treated with belatacept, 30 were HBsAg-negative and 5 (16.7%) of them reactivated HBV. This reactivation rate appears high. Indeed, HBV reactivation has been previously reported in several retrospective cohort studies focusing on kidney transplant recipients who are HBsAg-negative and HBcAb-positive; it was 0% in an American study including 49 patients,^12^ 2.7% in a Japanese study of 74 patients,^16^ 2.9% in a Portuguese study of 70 patients,^17^ 3.7% in an American study of 161 patients,^19^ 4% in a Korean study of 172 patients,^15^ 4.7% in a Chinese study of 322 patients,^14^ 6.5% in a Belgium study of 93 patients^13^ and 9.6% in a Japanese study of 52 patients.^18^

This study is the first, to the best of our knowledge, to focus on the risk of HBV reactivation in kidney transplant recipients receiving belatacept. It follows the observation previously reported of a belatacept-treated kidney transplant recipient that had presented with hepatitis secondary to HBV reactivation.^31^ The use of abatacept, another CTLA4 fusion protein, has been better documented, although outside of the transplantation setting. Abatacept differs from belatacept by 2 amino-acids and is used in the treatment of rheumatoid arthritis and psoriasis. In addition to 3 case reports of HBV reactivation,^32^, ^33^, ^34^ a retrospective study reported 3 HBV reactivations in a group of 29 HBsAg-negative HBcAb-negative patients who were treated with abatacept for rheumatoid arthritis.^35^ In contrast, Padovan *et al.*^36^ observed no reactivation in 72 HBcAb-positive patients treated with abatacept for rheumatoid arthritis after a 24-month follow-up. Altogether, abatacept treatment is considered at moderate risk (1%–10%) of HBV reactivation by the American Gastroenterological Association.^11^

Our conclusions regarding the high HBV reactivation rate with belatacept treatment cannot be definitive because of the absence of a control group in our study. Furthermore, most patients (91.9%) did not receive belatacept from transplantation, but treatments were initiated during follow-up because of low graft function for the majority. This may have induced bias because patients requiring changes in immunosuppression may be more susceptible to HBV reactivation, even though these changes were not related to over-immunosuppression. We believe that the chosen setting reflected best the use of belatacept in clinical practice.

This study failed to demonstrate a protective role for HBV antiviral prophylaxis. There may be several reasons for this. First, the small number of reactivations prevented effective statistical analysis. Second, patients receiving prophylaxis were most certainly the ones most at risk of reactivating HBV. Finally medication adherence was not monitored and patients who were prescribed prophylaxis may have not fully adhered to the treatment. Although the need for prophylaxis in HBsAg-positive kidney transplant recipients is well established,^20^ guidelines regarding HBsAg-negative HBcAb-positive patients have issued disparate recommendations. Whereas 2017 guidelines of the European Association for the Study of the Liver recommend no prophylaxis,^2^ the American Association for the Study of Liver Diseases states in its 2018 hepatitis B guidance that a prophylactic treatment of 6 to 12 months can be considered^37^; Kidney Disease: Improving Global Outcomes 2009 guidelines did not specifically address the issue of prophylaxis for HBsAg-negative patients but recommended a vaccination booster if HBsAb titer is <10 IU/l.^20^ Indeed, although antiviral prophylaxis for HBsAg-negative patients has proven to be beneficial in other populations at high risk of HBV reactivation such as patients receiving rituximab for hematological malignancies,^38^^,^^39^ in kidney transplant recipients there are no data except a retrospective study from Chen *et al.*^14^ that reported lamivudine prophylaxis as a protective factor of reactivation (odds ratio, 0.04). Given that risk of HBV reactivation appears higher with belatacept therapy and is close to reactivation rates reported with rituximab,^11^ it is our opinion that antiviral prophylaxis should be systematic for HBsAg-negative HBcAb-positive kidney transplant recipients receiving belatacept.

Another finding of the study was the similar patient and graft survival rates among HBcAb-positive and HBcAb-negative patients, which corroborates studies in the recent era that show similar outcomes for recipients with chronic HBV (HBsAg-positive patients)^3^ and for HBcAb-positive HBsAg-negative recipients,^40^ when compared with HBcAb-negative patients. However, we do not explain the 5-year eGFR difference between the 2 groups found in the present study. One hypothesis could be that geographic origin of recipients was different depending on HBcAb status, in accordance with endemic variations of HBV worldwide, with more patients of Sub-Saharan or Caribbean origin in the HBcAb-positive group (56.2% vs. 15.5% in the HBcAb-negative group). The use of the Modification of Diet in Renal Disease formula to determine eGFR and its high correction factor for race (1.21) may have overestimated eGFR in these patients and therefore explains this higher eGFR in the HBcAb-positive group.

In this study, HBV reactivation did not have a negative impact on patient or graft survival. Other studies in the transplantation setting have found contradicting results on the matter: Kanaan *et al.*^13^ reported no difference in patient and graft survival among a cohort including 6 reactivations whereas Chen *et al.*^14^ noted a lower patient survival among patients that reactivated HBV (*n* = 15) but no difference in graft survival. Larger studies are needed to better address this issue.

This study also tried to investigate the meaning of HBsAb loss during follow-up. If several studies have shown that presence of HBsAb at the time of transplantation was an important protective factor for HBV reactivation,^14^^,^^15^^,^^35^ the significance of HBsAb loss is less established. On a pathophysiological standpoint, HBsAb prevent the entry of HBV into hepatocytes and are thought to protect against HBV reactivation.^41^ Clinical data in the transplantation setting regarding loss of HBsAb preceding HBV reactivation consist primarily of case reports.^42^, ^43^, ^44^, ^45^ In other settings such as allogeneic hematopoietic stem cell transplantation, Onozawa *et al.*^46^ described 7 HBV reactivations out of 12 patients that had lost their HBsAb after stem cell transplantation, and in another cohort, out of 7 HBV reactivations, 5 had lost their HBsAb and the remaining 2 had HBsAb titers <15 IU/l.^47^ Considering these observations, we argue that attention should be paid to HBsAb disappearance during follow-up because it often precedes HBV reactivation. However, in our study 2 of the 7 reactivations occurred while HBsAb remained positive.

Finally, we noted the absence of HBV serologic follow-up for 2 of the 32 HBcAb-positive patients and of HBV PCR monitoring for 6 of them. Considering that the study was performed in 2 transplantation centers, conclusion on the quality of HBV monitoring after transplantation in general cannot be made. However, attention can be drawn to it, especially because we observed 2 cases of HBV reactivation that were not detected with serologic testing, in which HBV PCR became positive but HBsAg remained negative; we believe this emphasizes the importance of performing systematic HBV PCR during the follow-up of these patients. Furthermore, it may have underestimated the reported HBV reactivation rate, but reflected best clinical practice.

In conclusion, when compared with other studies in the kidney transplant setting, the rate of HBV reactivation in this cohort of HBcAb-positive patients treated with belatacept was remarkably high. These results are consistent with findings of increased rates of viral infections with belatacept treatment^24^, ^25^, ^26^, ^27^, ^28^^,^^30^ and should incline us to a particular attention toward prophylaxis and monitoring of HBV in these patients. Larger controlled studies are needed to confirm these findings and prospective trials should be carried out to determine the precise needs of antiviral prophylaxis in this population.

## Disclosure

All the authors declared no competing interests.
